# Supplementary material for: Genetic Diversity of Common Olive (Olea europaea L.) Cultivars from Nikita Botanical Gardens Collection Revealed Using RAD-Seq Method
Source: Genes (Basel). 2023 Jun 23;14(7):1323. doi: 10.3390/genes14071323 (PMC10379327; doi:10.3390/genes14071323)
Supplement: Supplementary file 1 [file genes-14-01323-s001.zip › Table S1.pdf]

Table S1. Samples of *Olea europaea* taken for analysis

№	Cultivar	Origin	Pedigree
1	Dalmatica	Dalmatia, former Yugoslavia	Old cultivar. Introduction
2	Ascolano	Italy	Old cultivar. Introduction
3	Gukates	Albania	Old cultivar. Introduction
4	Tiflisskaya	Caucasus	Old cultivar. Introduction
5	Nikitskaya	Crimea	The cultivar was found during the examination of plantations
6	Coreggiolo	Italy	Old cultivar. Introduction
7	Razzo	Italy	Old cultivar. Introduction
8	Leccino	Italy	Old cultivar. Introduction
9	Nikitskaya 2	Crimea	The cultivar was found during the examination of plantations
10	Obilnaya	Crimea	Coreggiolo x free pollination
11	Kolhoznitsa	Crimea	Coreggiolo x free pollination
12	Krymskaya Prevoskhodnaya	Crimea	Prevoskhodnaya x free pollination
13	Kalinjot	Albania	Old cultivar. Introduction
14	Pulazeqin	Albania	Old cultivar. Introduction
15	Karren	Albania	Old cultivar. Introduction
16	Della Madonna	Italy	Old cultivar. Introduction
17	Santa Caterina	Italy	Old cultivar. Introduction
18	Ofelankos	Spain	Old cultivar. Introduction
19	Bidza	Albania	Old cultivar. Introduction
20	Vajsi i Peqinit	Albania	Old cultivar. Introduction
21	Miskhorskaya	Crimea	The cultivar was found during the examination of plantations
22	Nikitskaya 37	Crimea	Perhaps Coreggiolo x free pollination

23	Nikitskaya 6	Crimea	The variety was found during the examination of plantations, possibly a relative of Tossiyskaya
24	Pikvales	Italy	Old cultivar. Introduction
25	Skorospelaya	Crimea	Coreggiolo x free pollination
26	Tossiyskaya	Caucasus	Old cultivar. Introduction. Parent plants unknown
27	Chernaya Rannyaya	Crimea	Coreggiolo x free pollination
28	'Old Olive Tree'	Crimea	Nothing is known, it has been growing on the southern coast of Crimea for over 2000 years
29	Nikitskaya 35	Crimea	The cultivar is selected among the plantations on the southern coast of Crimea
30	Nikitskaya 33	Crimea	The cultivar is selected among the plantations on the southern coast of Crimea
31	Nikitskaya 5	Crimea	The cultivar was found during the examination of plantations, possibly a relative of Tolgomskaya
32	Nikitskaya 29	Crimea	The cultivar is selected among the plantations on the southern coast of Crimea
33	D' Elemcen	North Africa	Imported from Abkhazia in 1901
34	Piangente	Italy	Imported from Italy in 1901
35	Tolgomskaya	Caucasus	Imported from Abkhazia in 1902
36	Otur	Caucasus	Unknown
37	Tavlinskaya	Caucasus	Received from the Artvinsky olive nursery of the Batumi region in 1910
38	Nisiot	Albania	Imported from Albania in 1954
39	Kokermadh Berati	Albania	Imported from Albania in 1954
40	D' Espagne	Spain	Imported from Abkhazia in 1901
41	Aglandau	France	Imported from Abkhazia in 1901

42	Lomashenskaya	Caucasus	Imported from Abkhazia in 1902
43	Nadzhvijskaya	Caucasus	Imported from Abkhazia in 1902
44	Gorvala	Caucasus	Received from the Artvinsky olive nursery of the Batumi region in 1902
45	Baki-8	Azerbaijan	Imported from Azerbaijan in 1952
46	Yubileinaya	Crimea	Selected among seedlings of the Rannyaya NBS cultivar, which was obtained by free pollination of the Coreggiolo cultivar
