# Supplementary figures and images for: Genetic Diversity of Common Olive (Olea europaea L.) Cultivars from Nikita Botanical Gardens Collection Revealed Using RAD-Seq Method
Source: Genes (Basel). 2023 Jun 23;14(7):1323. doi: 10.3390/genes14071323 (PMC10379327; doi:10.3390/genes14071323)

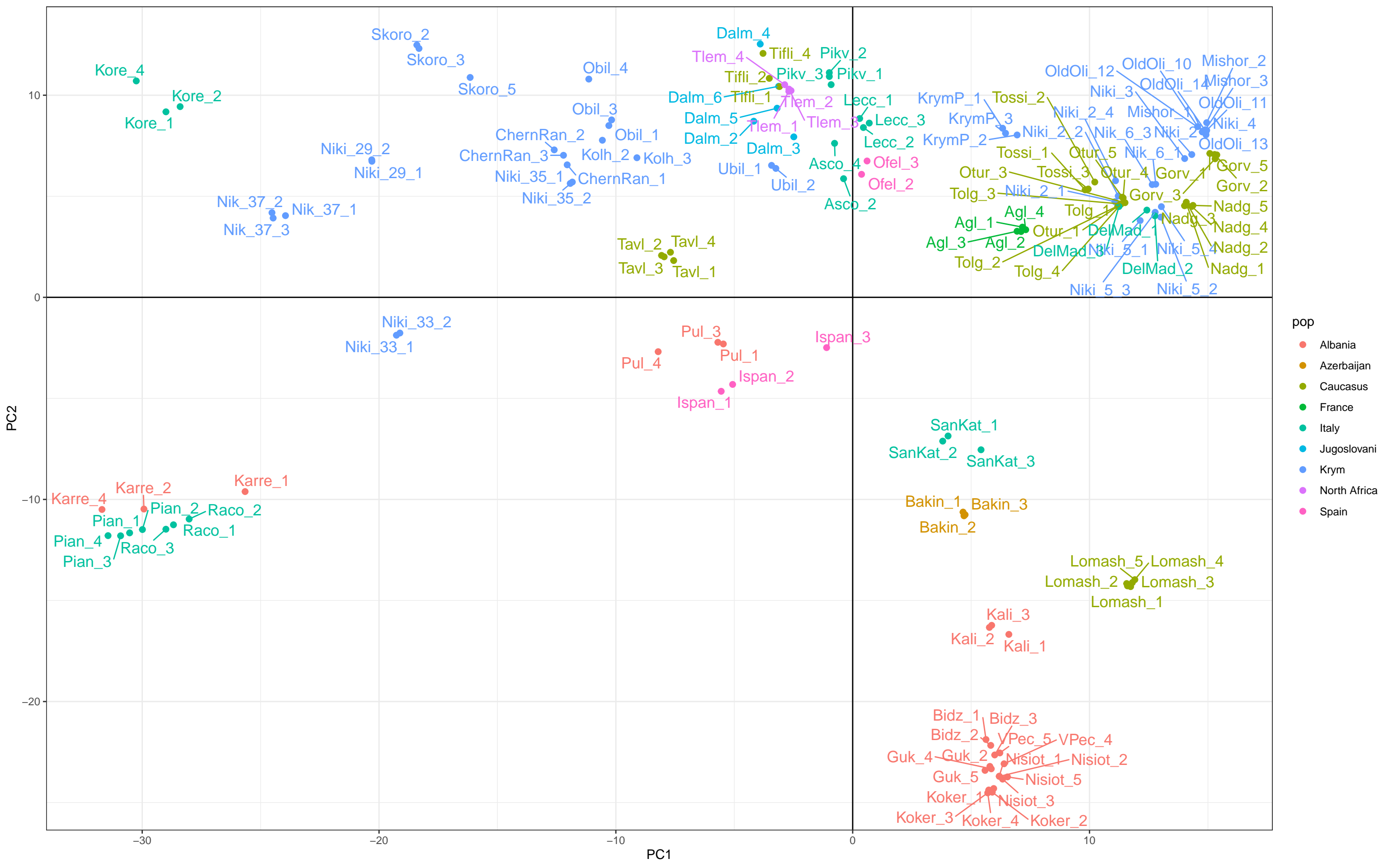

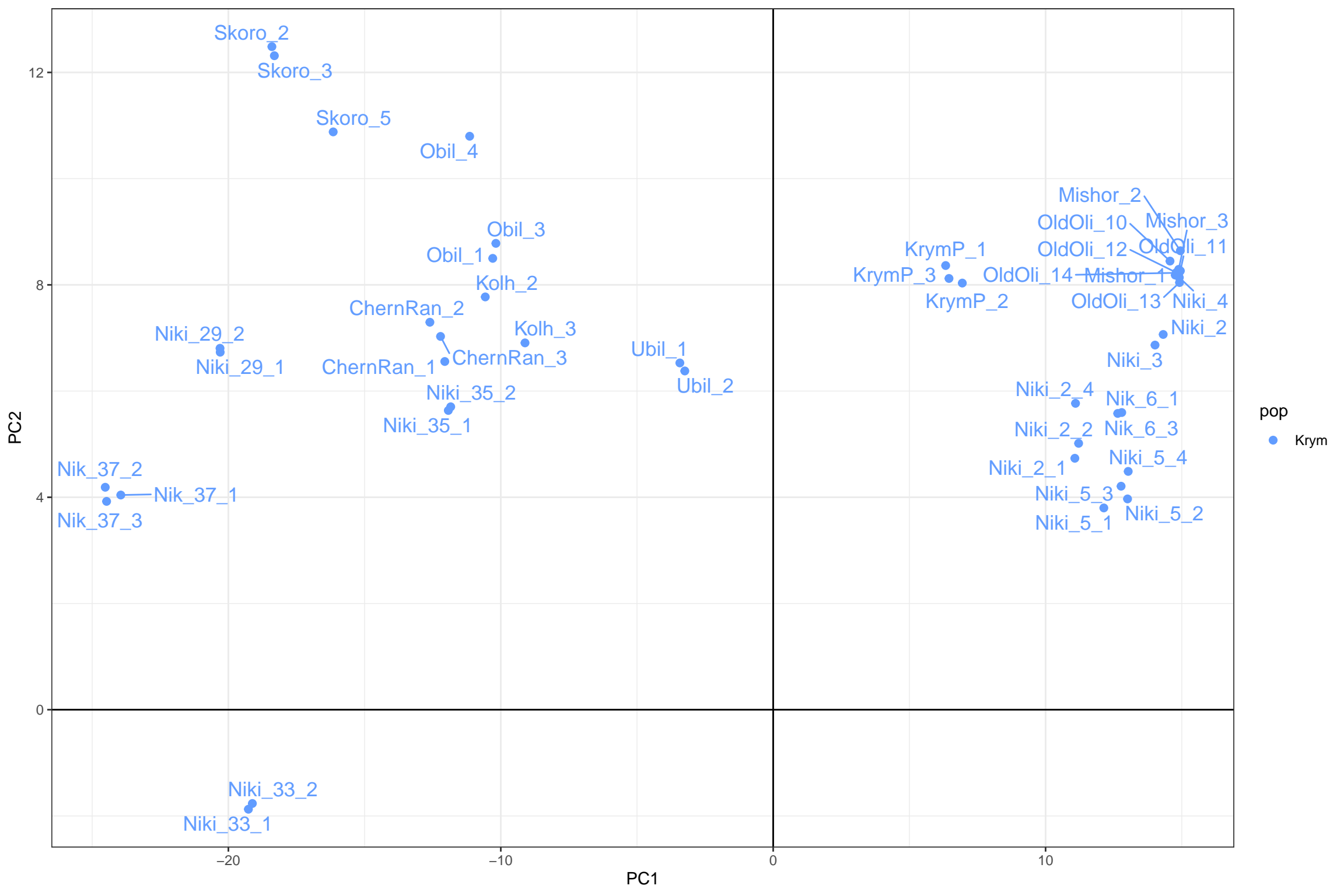

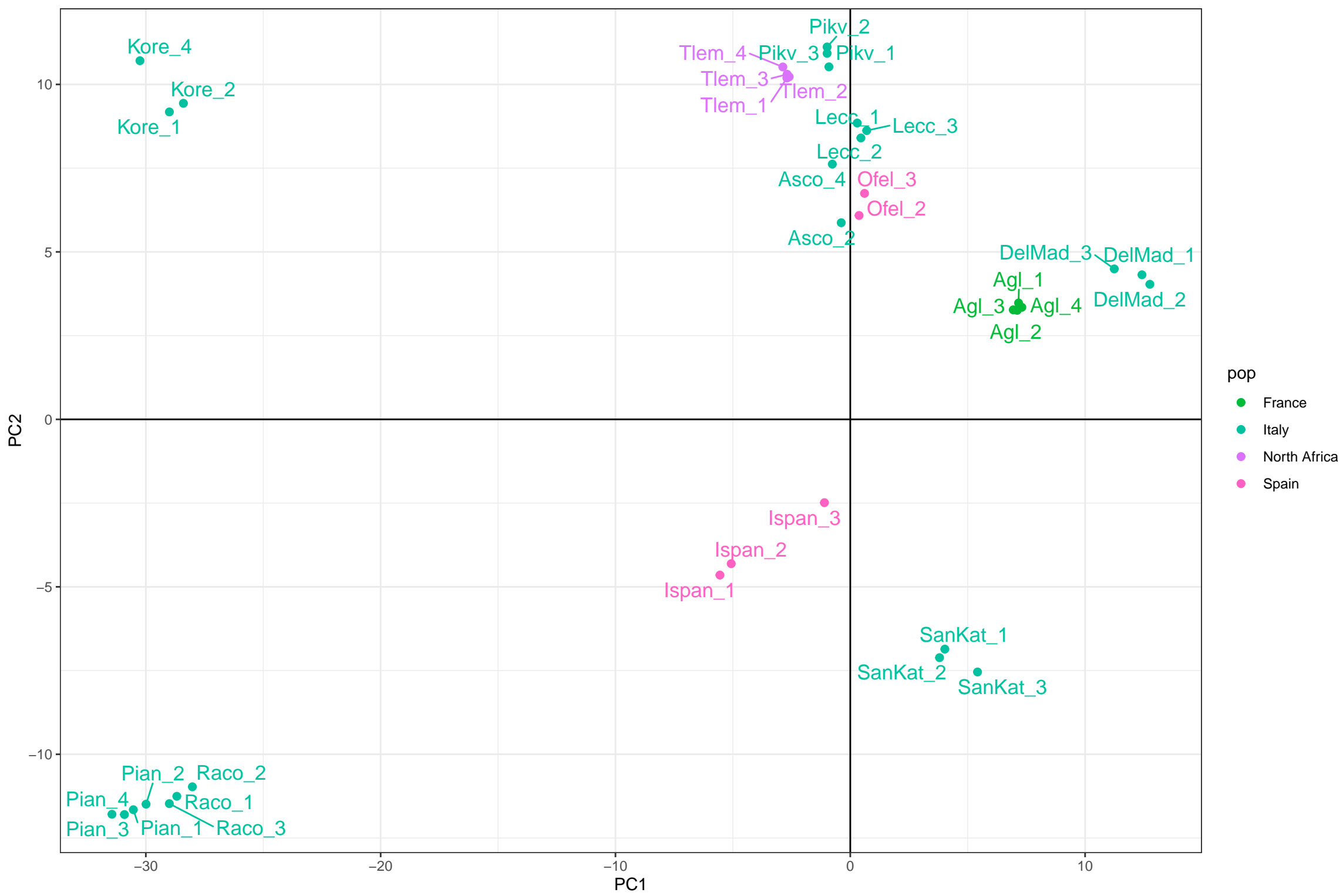

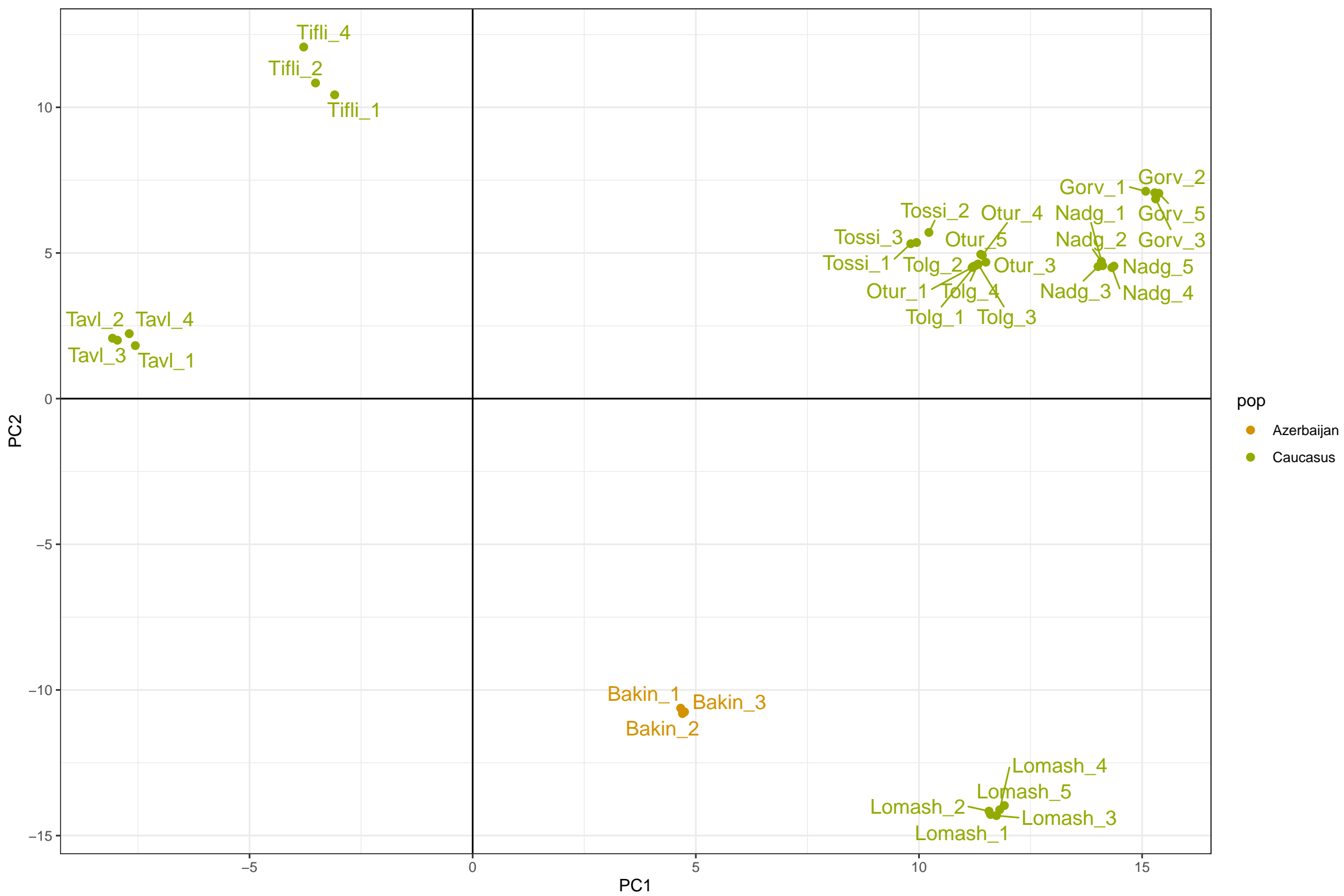

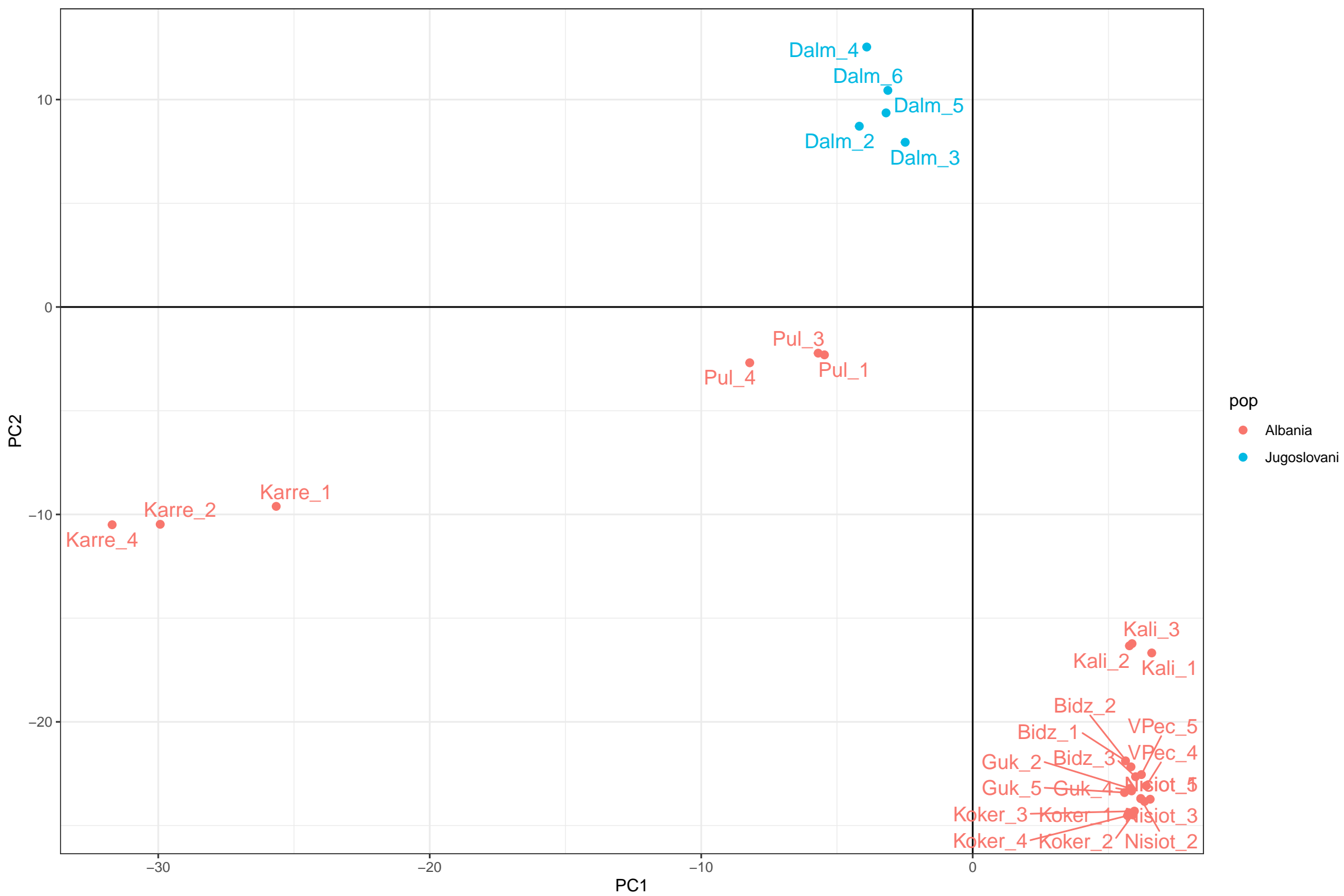

Supplement: Supplementary file 1 [file genes-14-01323-s001.zip › Figure S1.pdf]

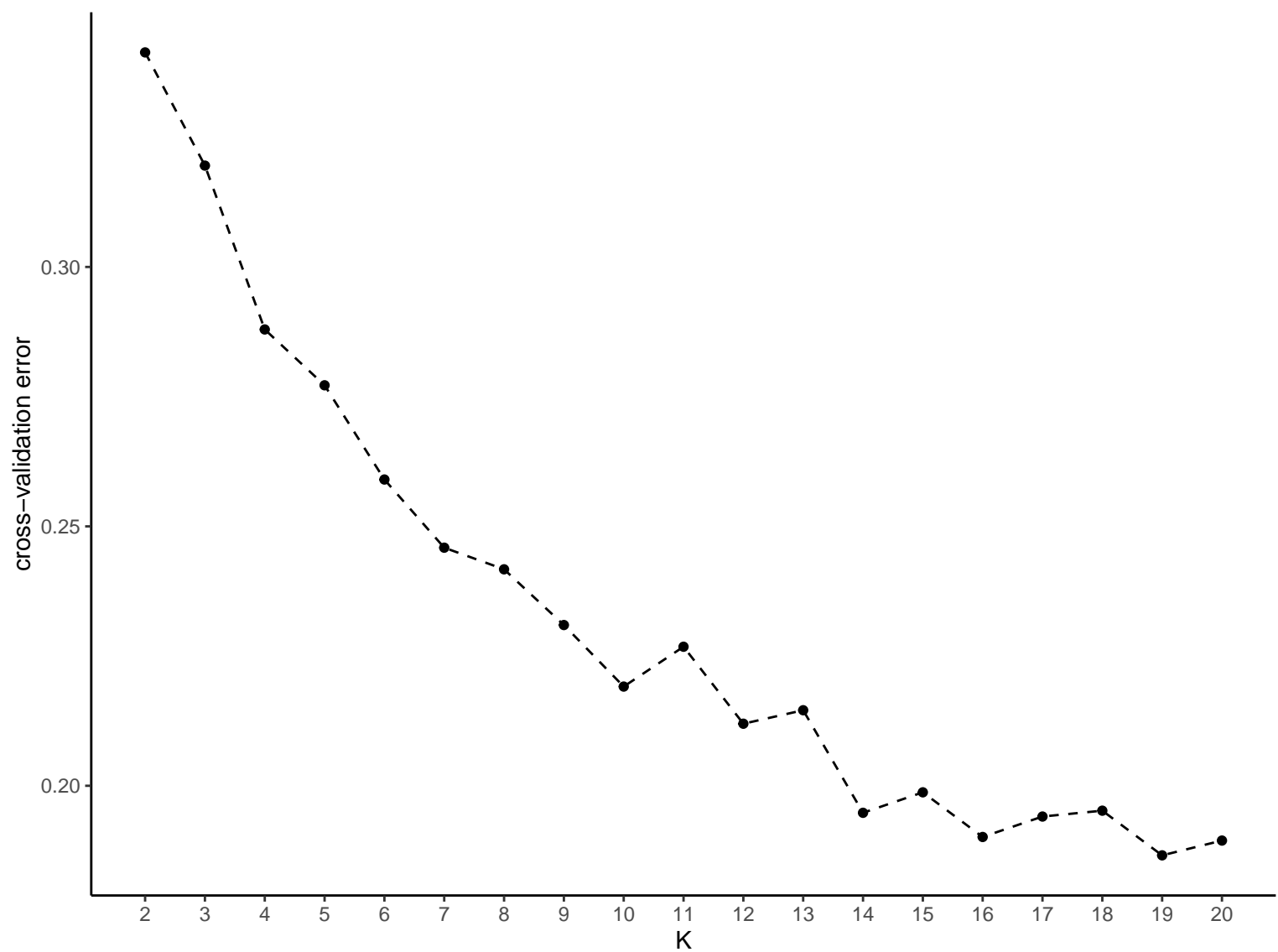

Supplement: Supplementary file 1 [file genes-14-01323-s001.zip › Figure S2.pdf]

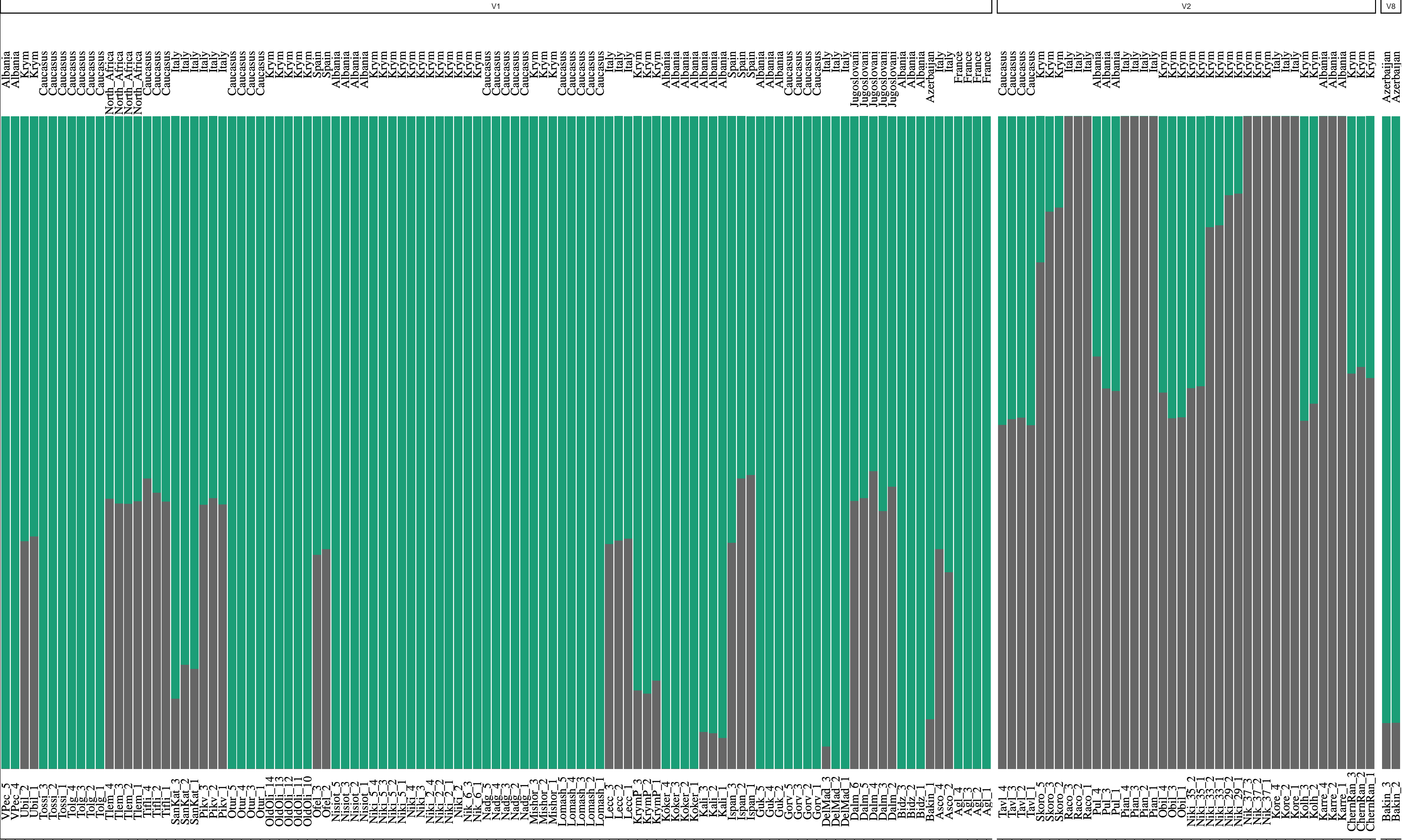



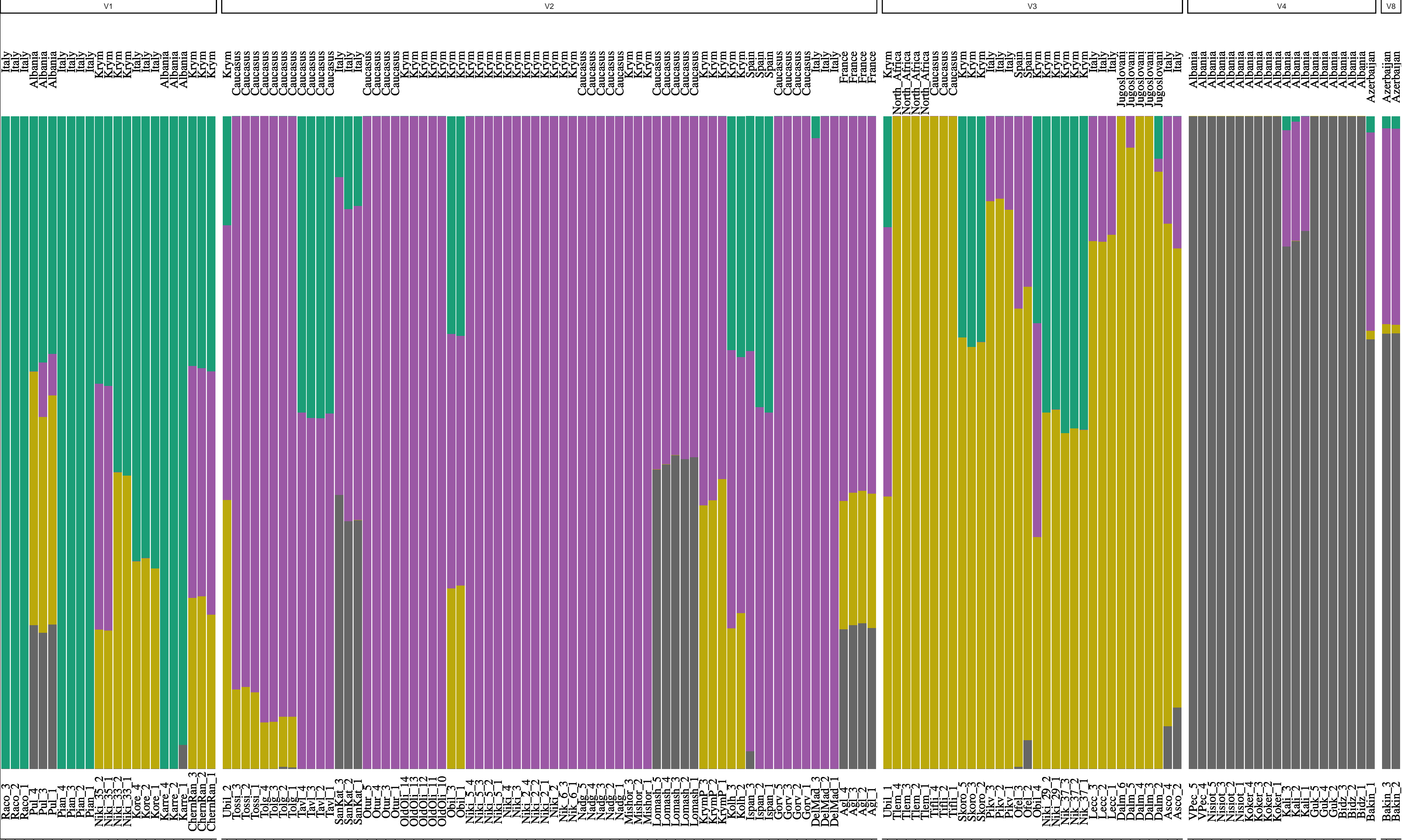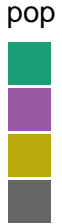

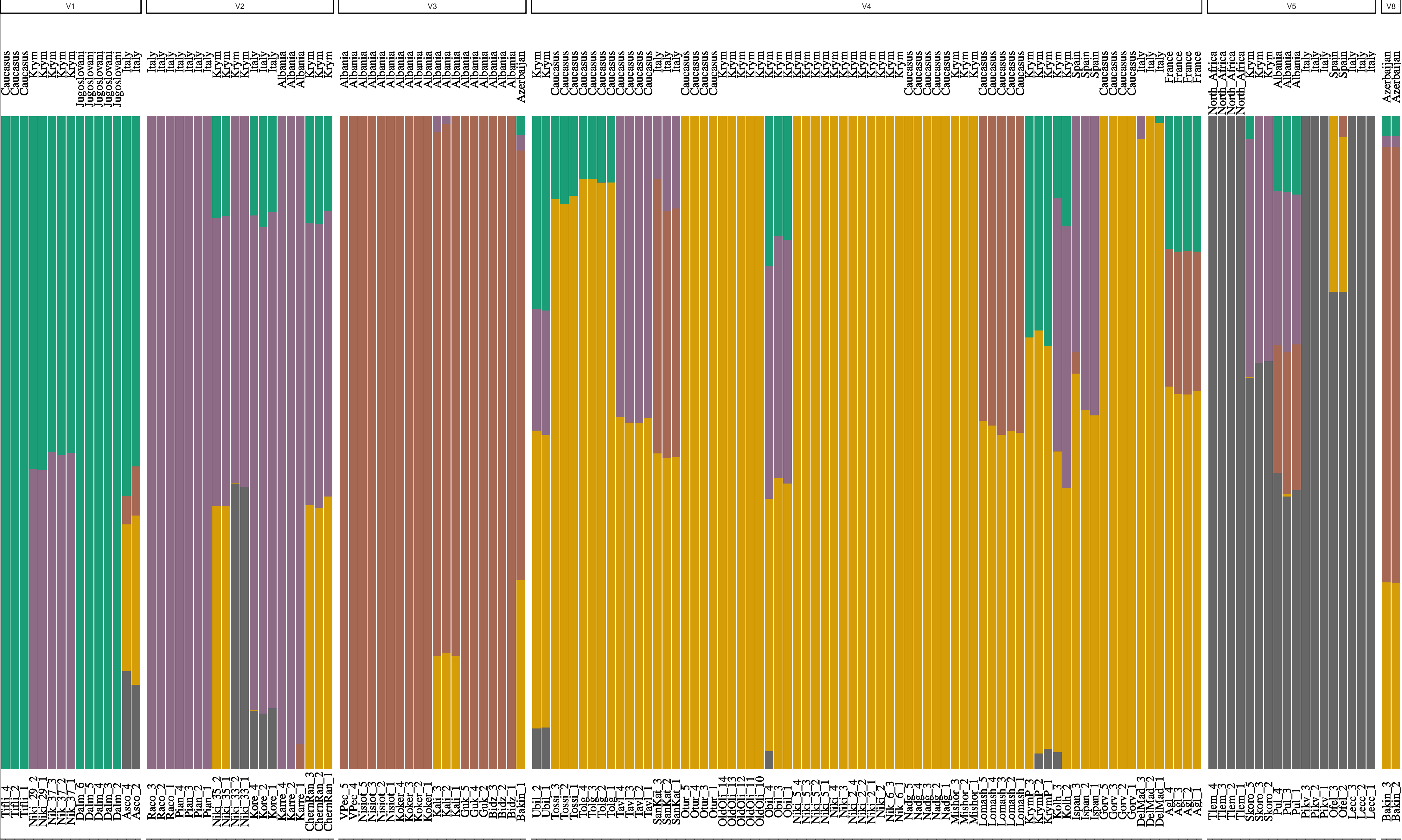

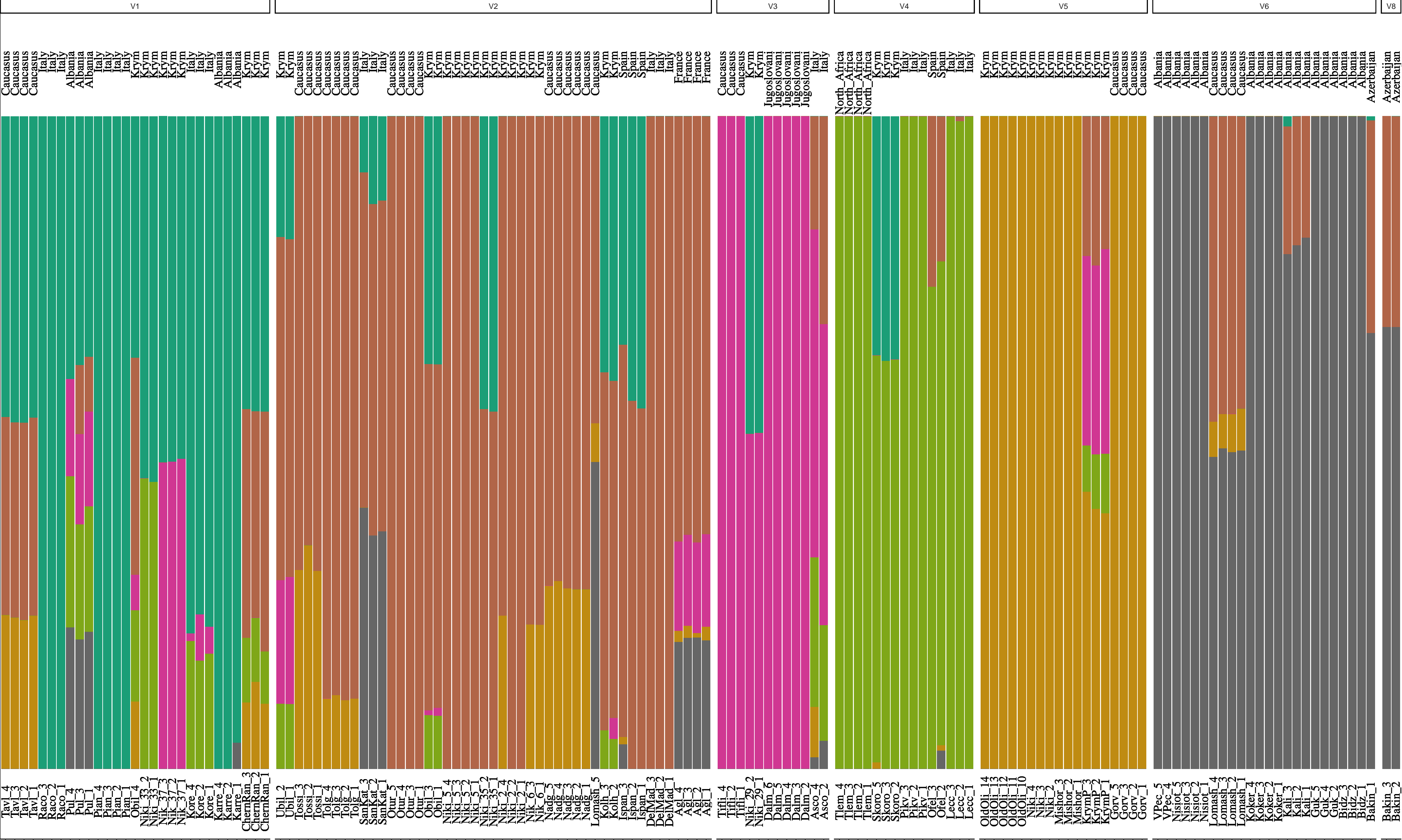











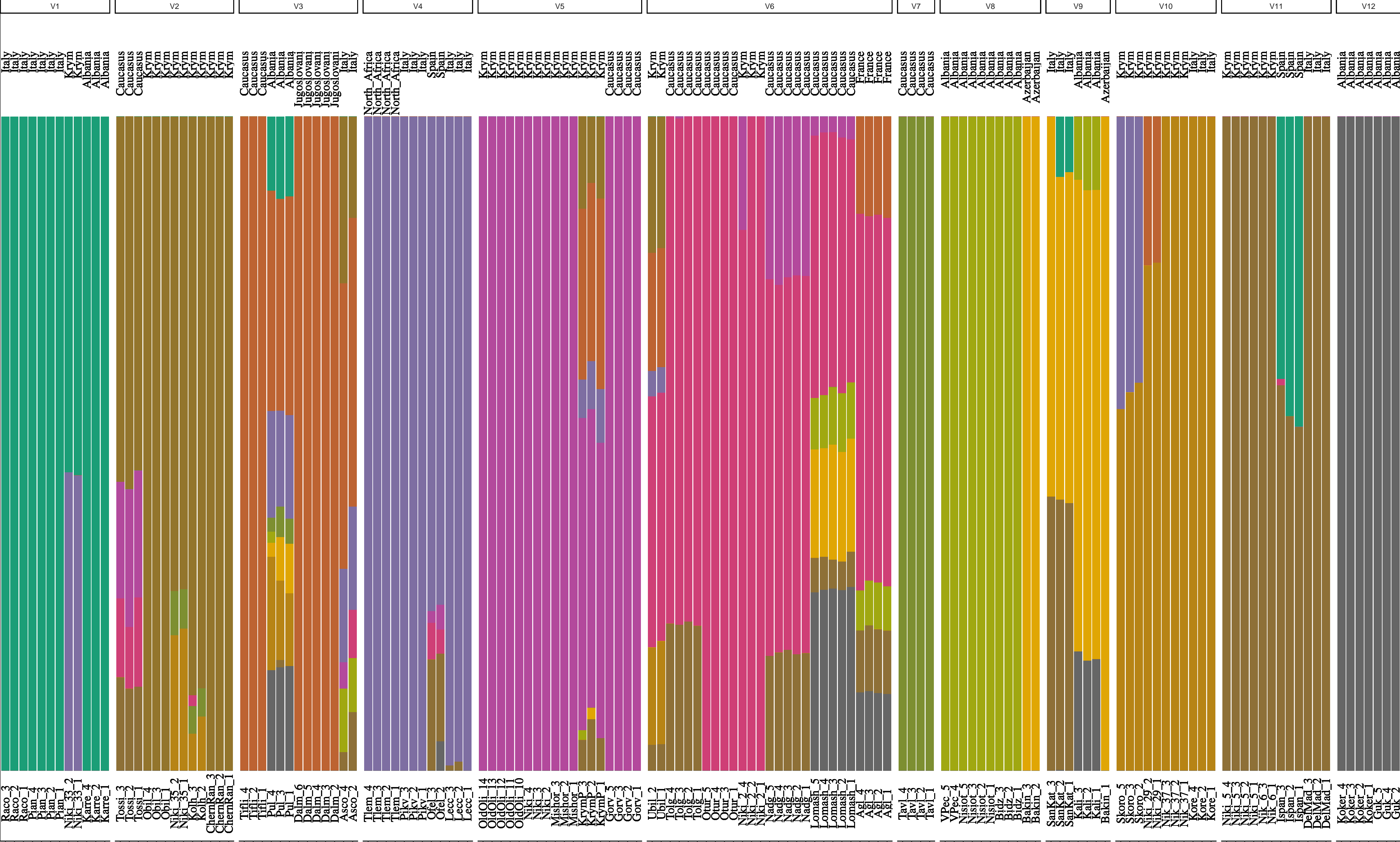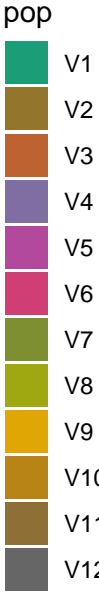





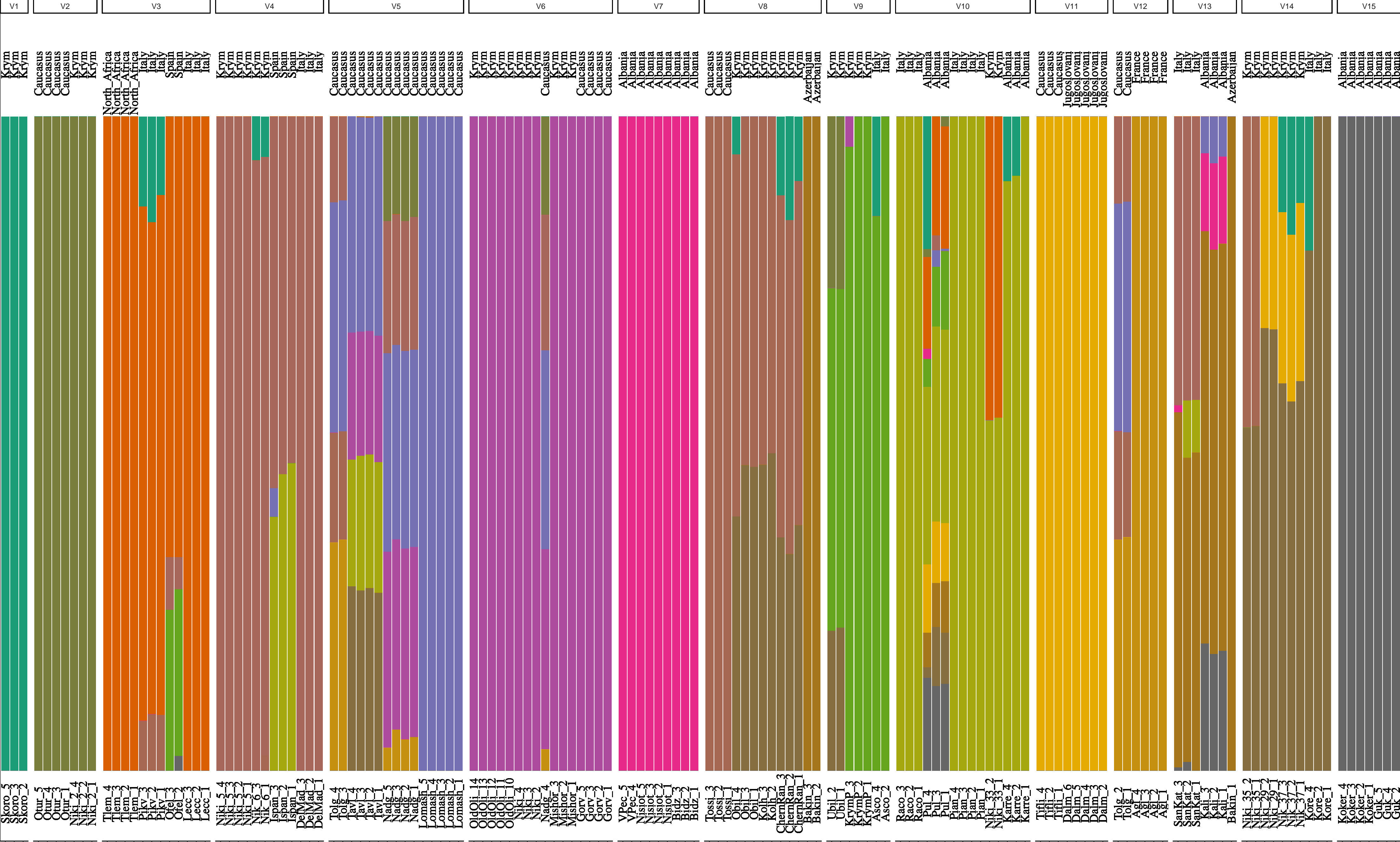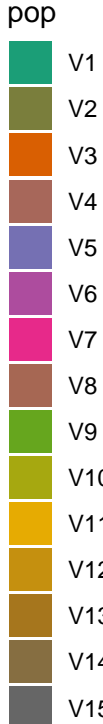







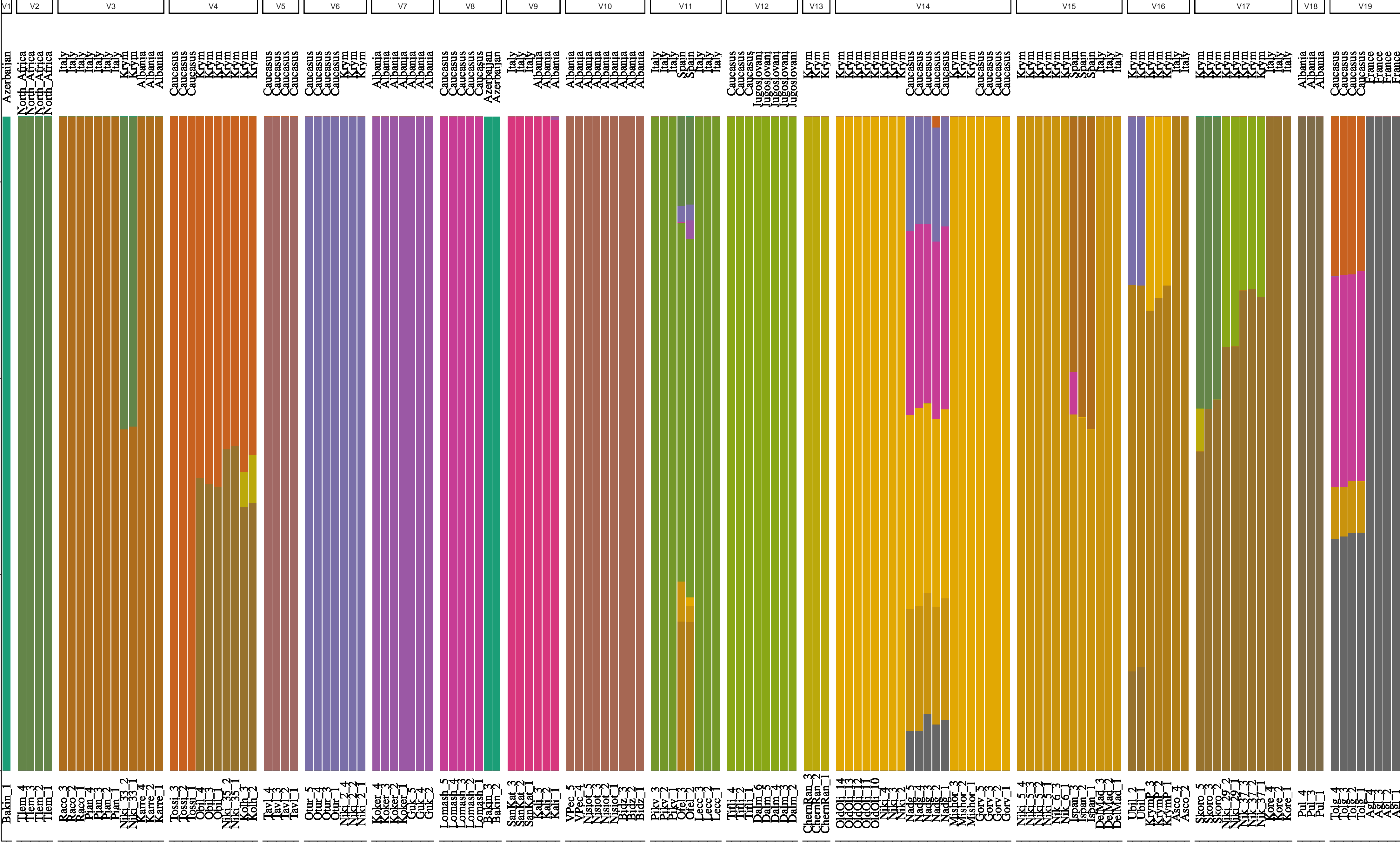

Supplement: Supplementary file 1 [file genes-14-01323-s001.zip › Figure S3.pdf]
