# Supplementary material for: Genetic Diversity of Common Olive (Olea europaea L.) Cultivars from Nikita Botanical Gardens Collection Revealed Using RAD-Seq Method
Source: Genes (Basel). 2023 Jun 23;14(7):1323. doi: 10.3390/genes14071323 (PMC10379327; doi:10.3390/genes14071323)
Supplement: Supplementary file 1 [file Table S2.pdf › Table S2.pdf]

Table S2. Phenotypic characteristics of some cultivars of olives

Cultivar	% of fat per wet weight	Fruit shape	Tree size	Bone/pulp ratio	Yield, kg	Leaf shape	Leaf length, mm	Leaf width, mm	Proportion of normal pollen grains, %	Fruit weight, g	Data of mass maturation
Nikitskaya 5	31	Round-oval, black peel	Vigorous, spherical crown	82:18	39–51	Oblong-lanceolate	80	15	87	5,4	01.11
Nikitskaya 6	30	Round-oval, black peel	Vigorous, spherical crown	85:15	40-50	Lanceolate	73	13	86	5,3	27.10
Della Madonna	30	Oval, black peel	Vigorous, spherical crown	85:15	38-46	Lanceolate	72	13	86	5,5	22.10
Tolgomskaya	31	Oval	Vigorous, spherical crown	82:18	39–51	Oblong-lanceolate	80	15	87	5,1	20.11
Tossiyskaya	30	Oval with pointed top	Vigorous, spherical crown	76:24<br>74:26	30-50	Lanceolate	73<br>70	13<br>10	86	5,4	30.10
Nikitskaya	36	Oval, black peel	Vigorous, spherical crown	82:18	25-40	Pointed-lanceolate	49	11	90	3,6	23.11
Miskhorskaya	26	Oval	Vigorous, wide-pyramidal crown	81:19	20-33	Lanceolate	50	11	88	3,0	22.11
Gorvala	28	Short oval or rounded, dark purple peel	Vigorous, vase-shaped crown	80:20	15-25	Lanceolate	52	13	90	6,0	24.11
'Old Olive Tree'	-	Oval	Medium height, widely spreading crown	80:20	-	Lanceolate	50	11	-	2,8	23.11
Tiflisskaya	24	Oval, black peel	Medium height, spherical crown	86:14	25-37	Broadly lanceolate	70	17	-	6,1	24.11
Dalmatica	24	Short oval, black peel	Medium height, widely spreading crown	84:16	27-34	Lanceolate	55	12	-	4,6	24.11

Razzo	33	Oval, tapered to the base, black peel	Vigorous, spherical crown	77:23	36-37	Lanceolate	55	12	-	3,3	02.12
Piangente	17	Oval	Vigorous, wide-pyramidal crown	80:20	30-34	Lanceolate	61	12	-	4,5	01.12
Bidza	26	Oval	Medium height, vase-shaped crown	82:18	22-29	Lanceolate	50	11	-	4,2	20.12
Vajsi i Peqinit	24	Oval	Vigorous, pyramidal crown	79:21	20-33	Lanceolate	56	12	-	4,9	08.11
Nikitskaya 2	32	Oval with pointed top	Vigorous, cupped crown	84:16	20-24	Pointed-lanceolate	74	13	95	6,5	18.10
Otur	32	Oval with pointed top	Vigorous, cupped crown	84:16	20-24	Pointed-lanceolate	74	13	95	6,4	20.10
